# A comprehensive analysis of the prevalence and influencing factors of felid alphaherpesvirus-1 (FHV-1) in mainland China from 2017 to 2024

**DOI:** 10.4142/jvs.25184

**Published:** 2026-06-19

**Authors:** Jiayu Shi, Huiling Zhang, Xinyuan Liu, Xueping Zhang, Hongyu Zhou, Tangjie Zhang

**Affiliations:** 1 Institute of Comparative Medicine, College of Veterinary Medicine, Yangzhou University, Yangzhou 225009, China.; 2 Jiangsu Co-innovation Center for Prevention and Control of Important Animal Infectious Diseases and Zoonoses, Yangzhou 225009, China.; 3 Independent Researcher, New York, NY 11355, USA.

**Keywords:** Alphaherpesvirinae, cats, mainland China, prevalence, meta-analysis

## Abstract

**Importance:**

Felid alphaherpesvirus 1 (FHV-1) is a common cause of feline upper respiratory disease, but prevalence estimates from mainland China are fragmented.

**Objectives:**

To estimate pooled FHV-1 prevalence and evaluate factors associated with between-study variation in mainland China from 2017 through 2024.

**Data Resources:**

PubMed, Web of Science, China National Knowledge Infrastructure, Wanfang Data, and the Chinese Journal Service Platform were searched; reference lists were also screened. Add the final search date.

**Study Selection:**

Cross-sectional studies from mainland China that reported a diagnostic method and sufficient numerator and denominator data were eligible. Two reviewers screened studies independently, with disagreements resolved by discussion or a third reviewer.

**Data Extraction and Synthesis:**

Thirty studies comprising 17,108 samples were included. Pooled prevalence was estimated with a random-effects model after Freeman-Tukey double-arcsine transformation. Heterogeneity, subgroup differences, publication bias, and leave-one-out sensitivity were assessed.

**Main Outcomes and Measures:**

Pooled FHV-1 prevalence with 95% confidence intervals (CIs), heterogeneity, and subgroup estimates by age, sex, breed, season, publication period, and region.

**Results:**

Overall pooled prevalence was 23% (95% CI, 18%–29%; I^2^ = 98.6%; heterogeneity *p* < 0.001). Prevalence was highest in cats aged 6 months or younger (51%; 95% CI, 26%–75%; subgroup *p* = 0.046) and in Exotic Shorthair cats (63%; 95% CI, 52%–74%; subgroup *p* = 0.003). Estimates differed by region (*p* = 0.021) but not by publication period (*p* = 0.742).

**Conclusions and Relevance:**

FHV-1 prevalence in mainland China was substantial and highly heterogeneous. Age, breed, and region were associated with variation across studies. Targeted prevention and surveillance may be warranted, but the very high heterogeneity and study-level subgroup analyses limit causal inference.

## INTRODUCTION

Felid alphaherpesvirus 1 or feline herpesvirus-1 (FHV-1), also known as feline viral rhinotracheitis (FVR), is classified within the order Herpesvirales, family Herpesviridae, subfamily Alphaherpesvirinae, and genus Varicellovirus. The virus is now officially called felid alphaherpesvirus-1 (commonly referred to as FHV-1) and is among the most common and highly contagious pathogens in cats [1]. It primarily affects the upper respiratory tract and eyes, causing symptoms such as sneezing, nasal discharge, conjunctivitis, and corneal ulcers. Severe cases can lead to skin ulcers and even death. FHV-1 poses a significant threat to young kittens, with an infection rate as high as 100% and approximately 50% mortality [2,3]. FHV-1 exhibits broad host susceptibility, a strong ability to establish a latent infection, and is prevalent worldwide, spreading rapidly [4,5]. The recovery period after an infection is prolonged, and some cats may become long-term carriers, continuously shedding the virus without showing clinical symptoms. This complicates any eradication effort and poses a sustained threat to domestic cats and wild felids [6,7]. In addition, the treatment costs for infected cats are high, and the protective efficacy of the currently available vaccines is limited. The high infection rate in catteries and animal shelters can lead to mass mortality or the production of unmarketable animals, further exacerbating economic losses [8,9].

With the growing pet cat population, the risk of FHV-1 infection has attracted increasing attention. China harbors one of the largest and most diverse feline populations worldwide, including household pets, stray cats, and cats in breeding or shelter facilities. FHV-1 is prevalent throughout China, posing significant health risks to domestic and wild felids [10,11,12,13]. Despite the recognized global importance of FHV-1, the epidemiological data from China remain fragmented and underrepresented in the international literature. Much of the available information is published in local Chinese journals, limiting accessibility for the broader scientific community. This knowledge gap hampers cross-regional comparisons and the development of globally relevant control strategies. Therefore, a critical evaluation of the existing epidemiological evidence from China is essential. The present study conducted a systematic review and meta-analysis of published data on FHV-1 in mainland China to provide a more robust estimate of its prevalence and risk factors. These results will support effective prevention and control strategies in China, contribute to the global understanding of FHV-1 epidemiology, facilitate vaccine development and diagnostic improvements, and safeguard the health of domestic and wild felids.

## METHODS

### Search strategy

Five bibliographic databases were searched, of which 2 were in English and 3 were in Chinese. The English PubMed and Web of Science search used “Feline viral rhinotracheitis” OR “Felid alphaherpesvirus 1” OR “FHV-1” AND “Epidemiology” OR “incidence” OR “prevalence”. The China National Knowledge Infrastructure, WanFang database, and Chinese Journal Service Platform were searched using the terms “Feline viral rhinotracheitis” OR “Felid alphaherpesvirus 1” OR “FHV-1” AND “Epidemiology” OR “incidence” OR “prevalence”. The reference list in each paper was searched for additional articles. All articles are obtained through library resources.

### Inclusion and exclusion criteria

Full-text articles were obtained and studies were included if they met the following criteria: 1) studies conducted in China, 2) studies involving the cat as the subject species, 3) cross - sectional study designs, 4) clearly described the corresponding detection methods, and 5) studies that included more than 10 clinical samples of FHV with specific data. Articles not meeting the inclusion criteria were excluded.

The following criteria were used: 1) literature reviews, 2) duplicate articles, 3) studies with fewer than 10 samples, and 4) studies conducted outside mainland China.

Literature screening was conducted independently by two researchers. When inconsistencies in the results occurred, discrepancies were resolved through discussion or by consulting a third reviewer.

The screened literature on cat prevalence was thoroughly reviewed, and the following information was extracted: author and publication year, sampling time and location, total number of samples collected, number of positive samples, and diagnostic methods used. Where necessary, the authors were contacted via email to obtain the necessary information. If the required data could not be obtained, the study was excluded from quantitative analysis but was retained for qualitative descriptions if relevant.

### Literature data extraction

The data extracted included the following: 1) study characteristics—the first author and year of publication; 2) study methodology—sampling location, time, and method; 3) characteristics of cats—age, sex, and breed; 4) sample size—the number of positives, and prevalence of FHV-1.

### Literature quality assessment

Cross-sectional studies in animals differ from other research designs, such as case-control studies, because the study population was not selected based on the dependent variable or outcomes. In cross-sectional studies, the participants were selected according to the inclusion and exclusion criteria established in the study, without accounting for exposure (independent variable) or outcome status (dependent variable) [14,15]. The systematic evaluation methods of animal cross-sectional clinical trials were adjusted based on the Cochrane quality evaluation, and the study quality was assessed by evaluating the following five items using RevMan 5.4 [16]. The scores were assigned according to a simple scale: 2 for “yes,” 1 for “not sure,” and 0 for “no.” 1) Are the research questions/objectives clearly described and stated? 2) Are the animal characteristics and sampling sites clearly reported? 3) Is the sampling method explained in detail? 4) Is the virus detection method clearly indicated? 5) Is the measurement outcome subject to subjective influence? Studies for which the total quality evaluation value was less than 6 were excluded.

### Statistical analysis

Stata 18.0 software was used for statistical analysis. First, I^2^ and Q tests were used to assess the heterogeneity in the effect size of each study [11], and the effect model was then selected; *p* ≥ 0.10 and I^2^ < 50% indicate low or no statistical heterogeneity between the effect sizes of each study. Accordingly, the inverse-variance model should be used for analysis in such cases; *p* < 0.10 and I^2^ ≥ 50% indicate large statistical heterogeneity between the effects of each study, and the DerSimonian–Laird model should be used. Meta-regression is used for different subgroups to estimate the odds ratios, determine whether it is a possible source of heterogeneity, and perform subgroup analyses of the possible causes of heterogeneity.

Although the inverse variance method is used widely and is suitable for 50% prevalence rates, the research will gain more weight when it is too small or large (0%–30% or 70%–100%), and other issues might arise. Therefore, such extreme values affect the final result and need to be adjusted to achieve a normal distribution as far as possible [17]. Therefore, before combining the prevalence estimates, the data were converted using the Freeman–Tukey double arcsine transformation (PFT) [18,19]. This transformation was selected because it stabilizes the variance when the prevalence values are close to 0% or 100%, thereby minimizing the influence of extreme proportions and ensuring more reliable pooled estimates. The PFT transformation formula is as follows:

t = arcsin [sqrt {r/(n + 1)}] + arcsin [sqrt {(r + 1)/(n + 1)}],

where t, n, and r represent the transformed detection rate, total sample size, and number of positive samples, respectively; se(t) = sqrt {1/(n + 1)}, where se = standard error, and the back transformation to a proportion was performed using: p = (sin(t/2))^2^.

The *p* value and I^2^ statistic were used to assess the heterogeneity among studies, guiding the selection of an appropriate effect model to estimate the effect size with a 95% confidence interval (CI). The heterogeneity was considered statistically insignificant when *p* ≥ 0.1 and I^2^ < 50%, and a fixed-effects model was applied accordingly. Conversely, significant heterogeneity was assumed when *p* < 0.1 and I^2^ ≥ 50%, and a random-effects model was thus used.

### Bias and sensitivity tests

A forest plot was generated to visualize the overall findings of the meta-analysis. The publication bias was assessed using a funnel plot and Egger’s test, with *p* values < 0.05 indicating significant bias, and *p* ≥ 0.05 suggesting a low risk of publication bias [20]. When asymmetry was detected in the funnel plot, the trim-and-fill method was used to estimate the number of potentially missing studies and calculate the adjusted prevalence accordingly [21,22,23]. Furthermore, sensitivity analysis was conducted to evaluate the robustness of the results [24], while subgroup analysis was performed to investigate the potential sources of the heterogeneity.

## RESULTS

### Literature search results

Three hundred and twelve relevant articles were identified through the search strategies applied to the five databases mentioned above. Following the literature screening process outlined in the Cochrane Handbook, the articles were sequentially evaluated based on their titles, abstracts, full texts, and exclusion criteria (Fig. 1A). Ultimately, 30 articles were included, comprising 25 Chinese studies and 5 English studies. These studies were used for the meta-analysis of the prevalence of FHV-1 in mainland China, totaling 17,108 cases. Supplementary Table 1 lists the data extracted from the 30 included studies, including the first author, year of publication, survey region, detection method, number of positive cases, and total number of samples.

**Fig. 1:**
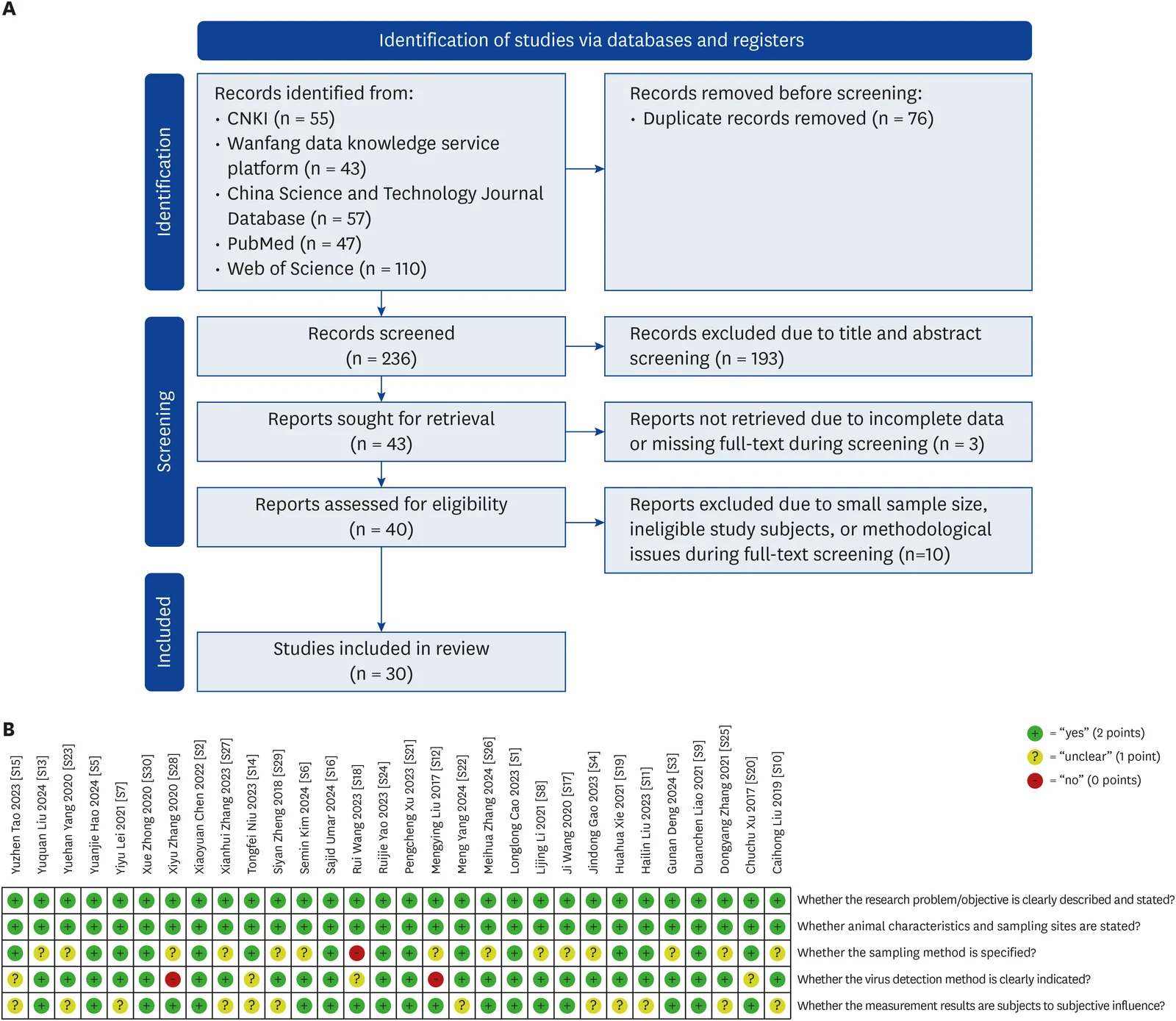
Study selection and quality assessment of the FHV-1 studies evaluated. (A) Flow diagram of the selection of eligible studies. (B) Quality assessment of studies screening on FHV-1. FHV-1, feline herpesvirus-1; CNKI, China National Knowledge Infrastructure.

### Baseline data included literature and quality assessment

The final 30 eligible studies covered central China. The studies were published from 2017 to 2024. The total number of cats was 17,108, with a range of 47–2,645. The detection methods were predominantly the polymerase chain reaction (PCR, n = 28).

According to the 5 quality assessment items, the total possible score was 10 points. The scores of the included studies ranged from 7 to 10, indicating high quality (Fig. 1B).

### Pooling and heterogeneity analyses

Double arcsine conversion was applied to the FHV-1 prevalence data reported in the 30 studies to estimate the FHV-1 prevalence and 95% CI. The heterogeneity index (I^2^) was 98.62% (χ^2^ = 2,005.47; *p* < 0.001; I^2^ = 98.6%), indicating that the 30 studies included in this meta-analysis were highly heterogeneous; Therefore, the random-effects model was applied to calculate the pooled prevalence. The meta-analysis showed that the incidence of FHV-1 was 23% (95% CI, 18%–29%), as shown in Fig. 2.

**Fig. 2:**
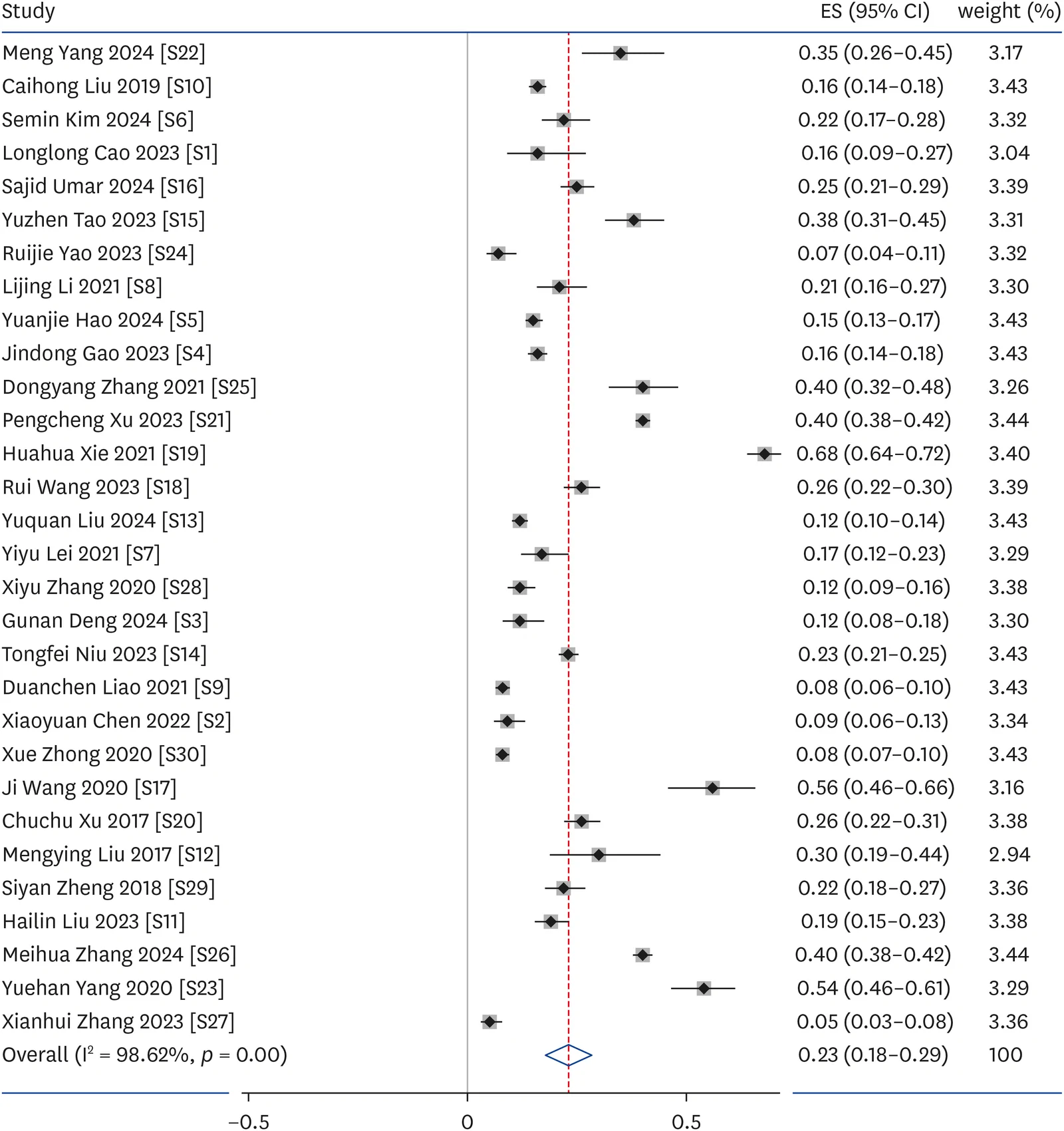
Forest plot of the prevalence of feline herpesvirus-1 with random-effects analysis. Squares show study prevalence estimates (area proportional to random-effects weight), horizontal lines show 95% CIs, and the diamond shows the pooled back-transformed prevalence. ES, effect size; CI, confidence interval.

Subgroup analyses were performed to identify the sources of heterogeneity in FHV-1 prevalence. Studies were stratified according to age, sex, breed, season, publication year, and region. A random-effects model (DerSimonian–Laird method) was applied to estimate the pooled prevalence within each subgroup. The results are presented in Table 1 and Supplementary Fig. 1.

**Table 1:** Subgroup analysis of felid alphaherpesvirus 1 prevalence and heterogeneity by potential associated

Variables		No. of studies included	No. of samples	No. of positive samples	Prevalence (%) (95% CI)	Heterogeneity			Subgroup-difference *p* value
						Heterogeneity: *p* value	I^2^ (%)	Q (χ^2^)	
Overall		30	17,108	4,213	23 (18–29)	< 0.001	98.60%	2,005.47	
Age (mon)									0.046
	≤ 6	4	1,491	684	51 (26–75)	< 0.001	98.60%	219.14	
	7–12	4	795	283	34 (26–42)	0.017	70.40%	10.13	
	> 12	9	1,293	283	22 (19–24)	< 0.001	94.00%	132.57	
Sex									0.805
	Male	10	3,977	1,165	30 (18–44)	< 0.001	98.63%	658.49	
	Female	10	2,792	805	28 (17–40)	< 0.001	97.32%	335.56	
Breed									0.003
	Ragdoll	3	340	91	34 (10–65)	ND			
	Exotic Shorthair	2	175	53	63 (52–74)	ND			
	British Shorthair	5	1,282	193	27 (8–50)	< 0.001	96.60%	117.69	
	American Shorthair	5	426	125	31 (13–53)	< 0.001	94.40%	60.56	
	Chinese domestic cat	4	674	125	32 (11–58)	< 0.001	95.35%	64.48	
	Other	4	447	76	29 (13–49)	< 0.001	84.16%	18.94	
Season									0.212
	Spring	6	772	165	20 (9–33)	< 0.001	91.74%	60.53	
	Summer	5	774	66	10 (6–14)	< 0.001	67.73%	12.40	
	Autumn	6	1,028	118	15 (9–23)	< 0.001	85.52%	34.52	
	Winter	6	2,976	150	16 (5–31)	< 0.001	97.31%	185.82	
Publication year									0.742
	2017–2019	4	2,079	402	22 (16–29)	< 0.001	87.36%	23.73	
	2020–2022	10	4,209	862	27 (14–43)	< 0.001	99.08%	975.94	
	2023–2024	16	10,820	2,949	21 (15–27)	< 0.001	98.26%	860.57	
Region									0.021
	Northwest region	2	351	70	17 (14–22)	ND			
	North region	11	7,699	2,475	29 (22–37)	< 0.001	97.77%	492.90	
	South region	8	5,939	1,163	23 (12–37)	< 0.001	99.22%	893.63	
	Qinghai-Tibet Plateau region	0	0	0					

No eligible studies were identified for the Qinghai-Tibet Plateau region.

CI, confidence interval; FHV-1, felid alphaherpesvirus 1; I^2^, percentage of total variation attributable to between-study heterogeneity; Q, Cochran Q statistic; χ^2^, chi-square statistic; ND, not determined because the number of studies was insufficient for reliable estimation of between-study heterogeneity.

Age subgroup analysis (Supplementary Fig. 1A) showed a marked decline in the prevalence of FHV-1 with increasing age. The positivity rate was 51% among cats aged six months or younger (95% CI, 26%–75%). This rate decreased to 34% (95% CI, 26%–42%) in cats aged six to twelve months, and further declined to 22% (95% CI, 19%–24%) in cats older than one year. The differences in FHV-1 positivity among these age groups were significant (*p* = 0.046).

Subgroup analysis according to the breed revealed significant differences in the FHV-1 positivity rates among the various breeds (*p* = 0.003; Supplementary Fig. 1D). Exotic Shorthair cats had the highest prevalence at 63% (95% CI, 52%–74%), followed by Ragdoll cats at 34% (95% CI, 10%–65%) and Chinese domestic cats at 32% (95% CI, 11%–58%). American Shorthair cats also showed a 31 prevalence % (95% CI, 13%–53%). British Shorthair cats and other breeds showed slightly lower prevalences, with British Shorthair cats at 27% (95% CI, 8%–50%) and other breeds at 29% (95% CI, 13%–49%).

No significant differences in FHV-1 positivity were observed among the seasonal subgroups (*p* = 0.212; Supplementary Fig. 1C). The highest detection rate was observed in spring at 20% (95% CI, 9%–33%), followed by winter at 16% (95% CI, 5%–31%). Lower prevalence rates were recorded in autumn and summer at 15% (95% CI, 9%–23%) and 10% (95% CI, 6%–14%), respectively. Similarly, no significant difference in FHV-1 positivity was observed between sexes (*p* = 0.805; Supplementary Fig. 1B). Males showed a 30% prevalence (95% CI, 18%–44%), while females exhibited a slightly lower rate at 28% (95% CI, 17%–40%).

In this study, a lag of approximately 1–2 years was commonly observed between the dates of sample collection and the corresponding publication dates. Subgroup analysis based on the publication year indicated that the estimated prevalence of FHV-1 infection was 22% (95% CI, 16%–29%; Supplementary Fig. 1E) for studies published between 2017 and 2019, 27% (95% CI, 14%–43%) for those articles published between 2020 and 2022, and 21% (95% CI, 15%–27%) for studies published from 2023 to 2024. These differences in prevalence across the three periods were not significant (*p* = 0.742). The apparent stability in FHV-1 prevalence across publication periods may reflect the consistent infection rates over time. Nevertheless, this pattern should be interpreted with caution because it could also be influenced by variations in sample sizes, geographic coverage, or reporting practices.

Mainland China was divided into four climatic zones according to the geographical location, latitude, and altitude. Nevertheless, the analysis was limited to the northwest, north, and south regions because no data were available from the Qinghai–Tibet region. Subgroup analysis according to the region showed that the FHV-1 positivity rate was 17% (95% CI, 14%–22%) in the northwest region, 23% (95% CI, 12%–37%) in the south and 29% (95% CI, 22%–37%) in the north, with significant differences among regions (*p* = 0.021; Supplementary Fig. 1F).

### Publication bias analysis and sensitivity analysis

A funnel plot was used to assess the publication bias in the included studies (Fig. 3A). The funnel plot appeared asymmetric; however, Egger’s regression test did not detect statistically significant small-study effects. (*p* = 0.084, *p* > 0.05; Fig. 3B) [21,23].

**Fig. 3:**
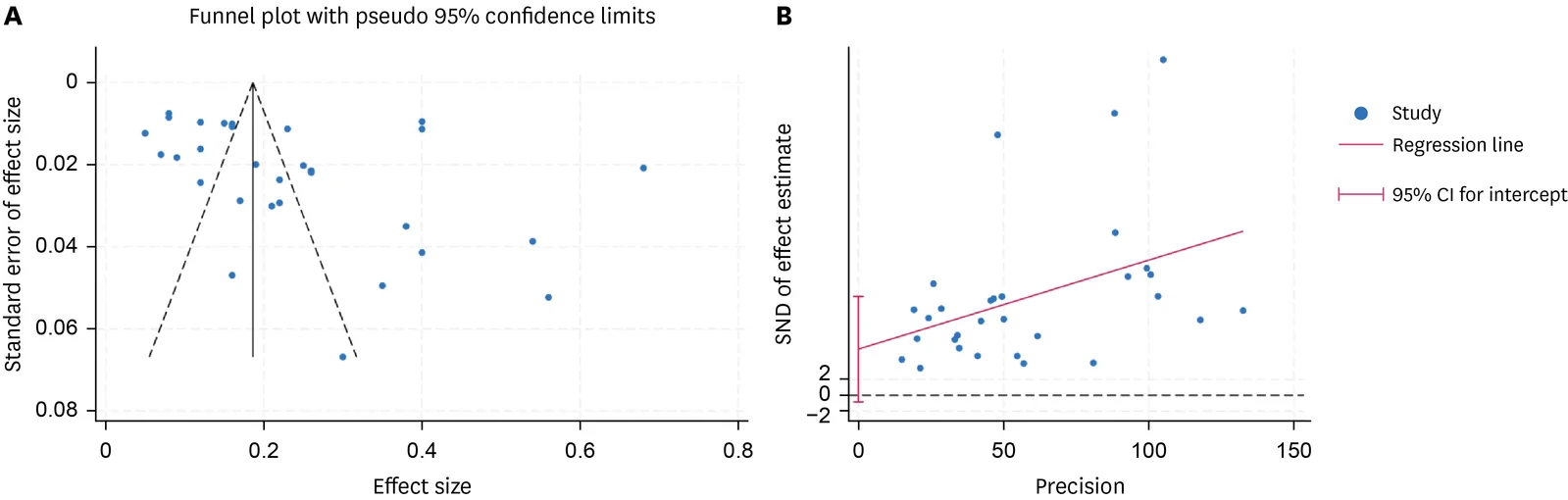
Assessment of publication bias in the meta-analysis of epidemiological studies on FHV-1. (A) Funnel plot of the meta-analysis of epidemiological studies on FHV-1. (B) Egger test of publication bias among epidemiological studies on FHV-1. The solid line represents the regression line, and the dotted lines indicate the 95% confidence interval boundaries. SND, standard normal deviate; FHV-1, feline herpesvirus-1; CI, confidence interval.

An iterative approach was used to estimate the number of missing studies based on the assumption that publication bias causes funnel plot asymmetry. A new meta-analysis was conducted using the trim-and-fill method (Metatrim command in Stata software) to assess the impact of publication bias on the results [21,22]. The meta-analysis was repeated after incorporating twelve additional imputed studies, yielding an adjusted *p* = 0.000 (Fig. 4A). The heterogeneity remained significant, suggesting that the corrected results were robust.

**Fig. 4:**
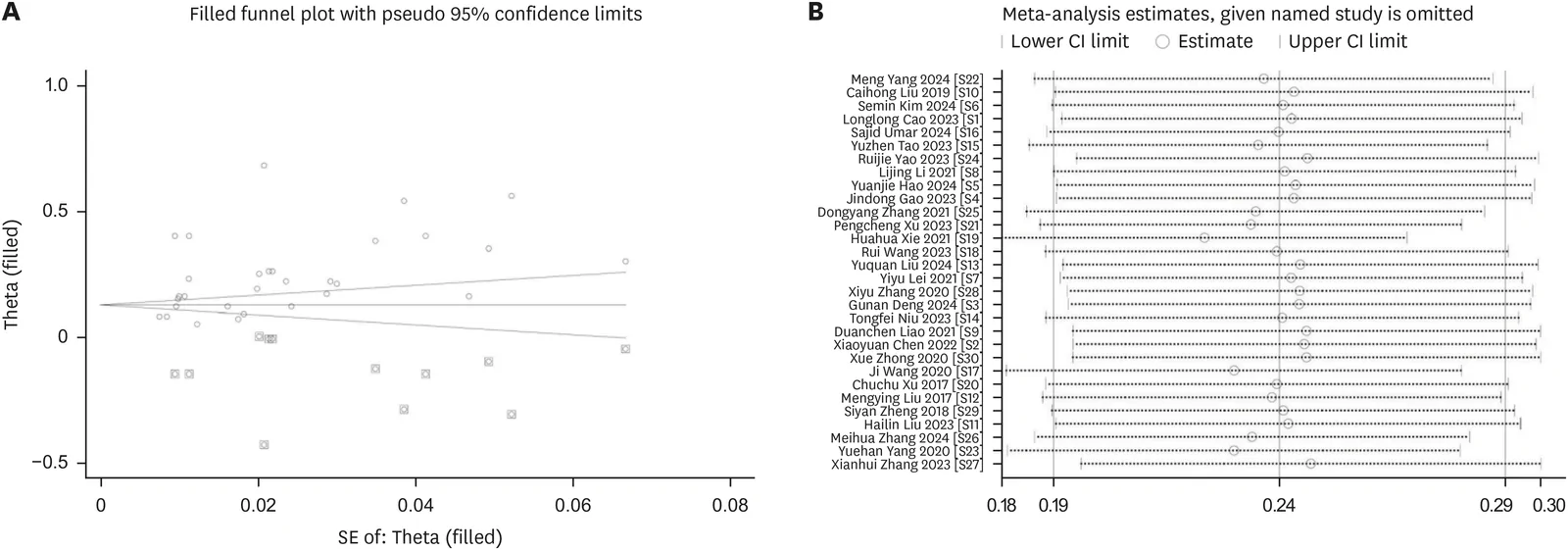
Publication bias adjustment and sensitivity analysis of epidemiological studies on FHV-1. (A) Filled funnel plot of meta-analysis with “trim-and-fill” method; circles indicate observed studies, and squares indicate trim-and-fill imputed studies. (B) Sensitivity analysis of epidemiological studies on FHV-1 (circles show pooled prevalence estimates after omission of each study, and horizontal bars show 95% CIs). FHV-1, feline herpesvirus-1; SE, standard error; CI, confidence interval.

The absence of robust sensitivity analysis methods can introduce uncertainty or lead to misinterpretation in research. Sensitivity analysis showed that the incidence ratio of FHV-1 remained within the 95% CI (Fig. 4B), suggesting that variations in the number of included studies had little effect on the meta-analysis results, confirming their robustness.

## DISCUSSION

FVR is a significant upper respiratory tract infection in cats with a worldwide distribution. Recent studies have reported varying prevalence rates across different countries. A 2023 study in the UK reported an FHV-1 prevalence of 2.1% based on buccal swabs from 430 pet cats [25]. A study conducted in Japan in the late 1990s reported a 3.6% prevalence among 111 cats with upper respiratory infections [26]. In Spain, the FHV-1 prevalence was 28.3% in cats with upper respiratory tract disease (URTD), while a control group of 98 healthy cats had a prevalence of 6.1% [27].

This study conducted a comprehensive epidemiological survey and performed a meta-analysis to estimate the pooled prevalence of FHV-1 and determine the associated risk factors for FHV-1 infection. Thirty eligible studies were included in the meta-analysis, and an epidemiological survey involving 17,108 samples was conducted, revealing an overall prevalence of FHV-1 of 23% (95% CI, 18%–29%) in mainland China. In this study, the observed prevalence of FHV-1 among cats with URTD was lower than the 25% to 50% prevalence typically reported in cats with acute URTD [28,29], but was consistent with the range reported by Sykes, who estimated an FHV-1 prevalence of 10.0%–34.0% in URTD-affected cats [30].

These findings suggest that cats of all age groups in mainland China are susceptible to FHV-1 infection, but significant differences in prevalence were observed across age groups (*p* < 0.05). The highest infection rate was noted in kittens aged six months or younger (51%), followed by those aged six to twelve months (35%); the lowest rate was observed in cats over one year of age (22%). These results revealed a clear decreasing trend in FHV-1 prevalence with increasing age. This age-associated pattern may be attributed to immunological and behavioral factors. Young kittens are particularly vulnerable to FHV-1 because of their immature immune systems, underscoring their heightened susceptibility to infection [1,2]. FHV-1 commonly causes acute upper respiratory disease, which is often severe in kittens between four and eight weeks of age—a critical period when the maternal antibody levels decline and active immunity has not yet developed. Prior to weaning, kittens rely primarily on the maternal antibodies transferred through colostrum for passive protection [31,32]. Compounding this vulnerability, queens are frequently latent carriers of FHV-1. Reactivation of the virus during breeding or lactation can lead to horizontal transmission and outbreaks within entire litters, especially in breeding catteries [33]. Although maternal antibodies provide initial protection, vaccinating the queen does not prevent viral reactivation or transmission to their offspring. Therefore, the observed decline in FHV-1 prevalence among older cats may be attributed to a combination of acquired immunity over time and decreased exposure to high-risk environments.

Most studies suggest that gender is not significantly associated with the FHV-1 infection rates [34]. In this study, male cats had a 30% infection rate, while female cats had a 28% infection rate, with no significant difference between the groups (Table 1). The higher number of male cats in this study reflects the gender preference in pet selection in mainland China, where male cats are more favored, resulting in an imbalanced gender ratio. According to the 2021 China Pet Consumption Trend White Paper, 64% of pet cats were male [35]. On the other hand, female stray cats face greater risks and higher mortality rates because of pregnancy and birth, which require more energy and nutrition [36]. Existing studies have not explicitly established a direct relationship between FHV-1 and sterilization. Although environmental stressors such as relocation and surgery are recognized as triggers for the reactivation of latent FHV-1, sterilization plays a crucial role in controlling the feline population, potentially mitigating the spread of FHV-1 in multi-cat environments [37]. Sterilization was not included in this study owing to the absence of relevant data.

The Exotic Shorthair cat showed the highest positive detection rate (63%) among the individuals sampled in this study. This elevated susceptibility may be attributed to its unique physiological structure, including a flat face, short and narrow nasal cavity, and restricted respiratory tract, which render it more susceptible to viral and bacterial infections [38]. Moreover, this observation may be associated with the inbreeding practices commonly involved in the breeding of purebred cats (such as Exotic Shorthair), leading to high genetic homozygosity, a weakened immune system, and increased susceptibility to epidemic viruses [39,40]. In addition, certain breeds, particularly those known to be more prone to stress, such as Siamese cats, may exhibit an increased risk of FHV-1 reactivation because stress is a well-known trigger for viral recurrence [41,42]. Therefore, the breed-related susceptibility to FHV-1 appears to be influenced by a combination of genetic and physiological factors.

Seasonal factors can also influence the FHV-1 infection rates, but this relationship varies depending on the environmental conditions and management practices. Several studies have suggested that FHV-1 infections may have increased prevalence during certain seasons because of a combination of factors, including temperature fluctuations, humidity, and changes in cat behavior [43,44]. In this study, the prevalence of FHV-1 infection was relatively low during the summer months (June to August), whereas a higher infection rate of approximately 20% was observed in spring (March to May). The infection rates in autumn (September to November) and winter (December to February) were comparable. Nevertheless, subgroup analysis revealed no significant differences in FHV-1 prevalence across seasons. Increased feline activity during spring and summer, along with the species’ characteristic of being seasonally polyestrous, with natural estrus and mating predominantly occurring in spring and autumn, may expose cats to more stressors and environmental factors, elevating the risk of FHV-1 infection [45,46]. In addition, the substantial diurnal temperature variation in spring and autumn can contribute to physiological stress and reduce the immune function, further increasing the susceptibility to infection [47].

The specific sampling times reported in the included studies varied because of differences in the publication and acceptance timelines. In most cases, however, the publication date was one to two years after the sampling period. The present study used the publication year as a proxy for comparing the FHV-1 prevalence across different time periods. The analysis based on the publication years showed no significant differences in FHV-1 prevalence among the three time periods. The consistently stable prevalence suggests that FHV-1 remains endemic in the population, likely due to the ongoing viral circulation under relatively balanced transmission dynamics and control measures.

The Northwestern, Northern, and Southern regions of mainland China differ in geography, latitude, and altitude, resulting in distinct climatic conditions that can affect the disease occurrence and transmission. The FHV-1 positivity was lowest in the Northwestern region, due likely to the sparse population, fewer domestic cats, limited veterinary services, and the small number of included studies. The prevalence in the Northern region was slightly higher than in the South, possibly reflecting the prolonged viral survival in colder climates. Most available data were derived from urban pet or stray populations, which may not fully represent rural cats, which typically experience weaker veterinary coverage and different management practices.

The aim was to assess the current epidemic status of feline FHV-1 in mainland China, contribute to global understanding of FHV-1 epidemiology, identify effective prevention and control strategies, monitor trends in viral transmission, support vaccine development and diagnostic advancements, and safeguard the health of both domestic cats and wild felids.

Regarding the detection methods, PCR has largely supplanted bacterial culture and virus isolation because of its higher sensitivity and faster turnaround time. In the present study, all but two studies, which did not specify the detection method, used PCR for FHV-1 identification. In addition, co-infection with feline calicivirus (FCV) is commonly reported in clinical settings, and a few included studies documented the concurrent detection of FHV-1 and FCV, highlighting the need for future investigations into the prevalence and clinical significance of such co-infections.

Given the widespread prevalence of FHV-1 and its significant impact on feline health, effective management strategies are essential. Vaccination reduces the severity and spread of both viruses but does not always prevent infection or viral shedding. Nevertheless, it is unclear to what extent differences in vaccine coverage might have influenced the observed prevalence estimates because vaccination status was unavailable in the included studies. There is no curative treatment for FHV-1. Management typically includes supportive care, antiviral agents (e.g., famciclovir for FHV-1), and symptomatic treatment.

This study had several limitations. First, most samples were obtained from pet or stray cats in urban areas, whereas rural cat populations were underrepresented. This underrepresentation may bias national prevalence estimates because rural cats are often free-roaming with limited access to vaccination and veterinary care, potentially leading to higher infection rates and broader transmission within local communities. Second, the vaccination status was unavailable in the included studies. The absence of such data limits the interpretation of prevalence estimates because vaccination can reduce the disease severity and viral shedding. Incompletely vaccinated or unvaccinated cats may disproportionately contribute to the persistence of FHV-1, and future studies should integrate vaccination records to allow for stratified analyses. Third, FCV and FHV-1 are recognized as the most common viral agents of URTD, and co-infections are relatively frequent in clinical practice. Although only a few of the included studies mentioned the co-infection rates, insufficient data precluded quantitative analysis. Future epidemiological investigations should assess the co-infection patterns to clarify their role in disease dynamics.

## SUPPLEMENTARY MATERIALS

Supplementary Table 1 Basic data of the included literature

Supplementary Reference

Supplementary Fig. 1 Forest plot of the FHV-1 estimated incidence subgroup with random effects analyses. (A) The subgroup analysis of different ages. (B) The subgroup analysis of different sexes. (C) The subgroup analysis of different seasons. (D) The subgroup analysis of different breeds. (E) The subgroup analysis of different publication years. (F) The subgroup analysis of different regions.
