# Supplementary material for: A comprehensive analysis of the prevalence and influencing factors of felid alphaherpesvirus-1 (FHV-1) in mainland China from 2017 to 2024
Source: J Vet Sci. 2026 Jun 19;27(5):e46. doi: 10.4142/jvs.25184 (PMC13655567; doi:10.4142/jvs.25184)
Supplement: Supplementary file 1 [file jvs-27-e46-s002.doc]

**Supplementary Reference**

S1. Cao L, Chen Q, Ye Z, Li J, Zhang Y, Wang Y, et al. Epidemiological survey of feline viral infectious diseases in China from 2018 to 2020. *Anim Res One Health*. 2023;1(2):233-241.

S2. Chen X. *Epidemiological Investigation of Cat Infectious Diseases and Molecular Evolution of Feline Coronavirus in Parts of Guangdong Province*. Dissertation. Guangzhou: South China Agriculture University; 2022. https://d.wanfangdata.com.cn/thesis/D02715220

S3. Deng G, Zhang J, Bao Z, Chen T, Yu Q, Ding L, et al. Detection of feline herpesvirus type 1 and pathogenicity of an isolated strain. *Acta Vet Zootech Sin*. 2024;55(5):2253-2258.

S4. Gao J. Epidemiological investigation of upper respiratory tract infections in cats in Wuhan and the establishment and application of an FCV infection model. *Vet. Sci*. 2023;10: 46.

S5. Hao Y. *Epidemiological survey of feline viral rhinotracheitis in Xi’an and preliminary evaluation of inactivated vaccine protection*. Dissertation. Xianyang: Northwest A&F University; 2024. 10.27409/d.cnki.gxbnu.2024.002677.

S6. Kim S, Cheng Y, Fang Z, Zhongqi Q, Weidong Y, Yilmaz A, et al. First report of molecular epidemiology and phylogenetic characteristics of feline herpesvirus (FHV-1) from naturally infected cats in Kunshan, China. *Virol J*. 2024;21(1):115.

S7.Lei Y. *Development and application of common pathogen detection methods for upper respiratory tract infections in cats*. Dissertation. Guiyang: Guizhou University; 2021. 10.27047/d.cnki.ggudu.2021.001679

S8. Li L. *Molecular epidemiological investigation of feline herpesvirus type I and feline parvovirus infection in Southwest China*. Dissertation. Chengdu: Southwest Minzu University; 2021. 10.27417/d.cnki.gxnmc.2021.000170

S9. Liao D. *Epidemiological investigation of upper respiratory tract diseases in cats and evaluation of treatment methods in Guangzhou*. Dissertation. Guangzhou: South China Agriculture University; 2021. 10.27152/d.cnki.ghanu.2021.001138

S10. Liu C, Liu Y, Qian P, Cao Y, Wang J, Sun C, et al. Molecular and serological investigation of cat viral infectious diseases in China from 2016 to 2019. *Transbound Emerg Dis*. 2020;67(6):2329-2335.

S11. Liu H. Epidemiological survey and typical case analysis of upper respiratory tract diseases in cats in Beijing. *Mod J Anim Husb Vet Med*. 2023;44(7):45-48.

S12. Liu M, Li H, Shao M, Zhang W. Epidemiological investigation of upper respiratory tract diseases caused by FHV and FCV in cats in Beijing from autumn 2016 to spring 2017. *Chin J Vet Med*. 2017;39(4):73-75.

S13. Liu Y. *Retrospective analysis of feline herpesvirus infection in Shandong and exploration of the efficacy of Shuanghuanglian*. Dissertation. Taian:

53m Shandong Agriculture University; 2024. 10.27277/d.cnki.gsdnu.2024.000165

S14. Niu T. *Epidemiological survey and disease diagnosis of five upper respiratory pathogens in cats in Kunming City*. Dissertation. Hohhot: Inner Mongolia Agriculture University; 2023. 10.27229/d.cnki.gnmnu.2023.001968

S15. Tao Y, Liu Y, Yang J. Epidemiological investigation of feline herpesvirus and parvovirus in a pet hospital in Zhengzhou City. *J Tianjin Agric Univ*. 2023;30(3):59-62.

S16. Umar S, Muhammad S, Mouzahim M, Pleva SM, Zhongqi Q, Weidong Y, et al. Burden of common respiratory pathogens among cats in China. *Vet Med Sci*. 2025;11(1):e70082.

S17. Wang J, Fu R, Wang S, Feng Y, Wang S, Li W, et al. Investigation of feline herpesvirus type I infection in Beijing and Hebei. *Chin J Virol*. 2020;10(5):364-368.

S18. Wang R. *Epidemiological survey of upper respiratory tract diseases in cats in Shandong Province and VP1 gene sequence analysis of parvovirus*. Dissertation. Taian: Shandong Agriculture University; 2023. 10.27277/d.cnki.gsdnu.2023.000187

S19. Xie H. *Epidemiological survey of feline herpesvirus and parvovirus infection in Shanghai and evaluation of treatment efficacy*. Dissertation. Yinchuan: Ningxia University; 2021. 10.27257/d.cnki.gnxhc.2021.001523

S20. Xu C, Hu S, Chen S, Liu C, Liu Y, Lü Y. Epidemiological investigation of feline parvovirus and herpesvirus in Beijing. *Chin Vet J*. 2017;53(11):23-26.

S21. Xu P. *Epidemiological investigation and treatment trials of feline infectious rhinotracheitis and calicivirus in Shenyang*. Dissertation. Shenyang: Shenyang Agriculture University; 2023. https://doi.org/10.27327/d.cnki.gshnu.2023.001053

S22. Yang M, Mu B, Ma H, Xue H, Song Y, Zhu K, et al. The latest prevalence, isolation, and molecular characteristics of feline herpesvirus type 1 in Yanji City, China. *Vet Sci*. 2024;11(9):417.

S23.Yang Y. *Epidemiological investigation and analysis of feline herpesvirus type I and feline parvovirus in Anhui Province*. Dissertation. Hefei: Anhui Agriculture University; 2020. 10.26919/d.cnki.gannu.2020.000194

S24. Yao R. Investigation and analysis of feline herpesvirus disease prevalence in Southern Xinjiang. Dissertation. Aral: Tarim Agriculture University; 2024. 10.27708/d.cnki.gtlmd.2024.000464

S25. Zhang D. *Investigation of upper respiratory tract disease in cats in Urumqi and analysis of typical case*. Dissertation. Ürümqi: Xinjiang Agriculture University; 2021. 10.27431/d.cnki.gxnyu.2021.000737

S26. Zhang M, Zhang Z, Lu F, Zhu F, Zhao J, Su Y, et al. Epidemiological investigation of feline panleukopenia virus, herpesvirus and parvovirus in cats in Beijing. *Chin Vet J*. 2024;60(8):138-141.

S27. Zhang X, Xiao Y, Liu C, Jiang Q, Zhang W, Sun Q, et al. Investigation and analysis of common pathogens of upper respiratory infections in cats in Chengdu in 2021. *Mod Anim Husb Sci Technol*. 2023;8:36-38.

S28. Zhang X. *Development and Application of Real-Time PCR Methods for Three Feline Susceptible Viruses*. Dissertation. Nanjing: Nanjing Agriculture University; 2020. 10.27244/d.cnki.gnjnu.2020.002141

S29. Zheng S, Lü Y, Xie Q. Investigation of major pathogens in 306 cases of upper respiratory infections in Cats in Beijing. *Chin Vet J*. 2018;54(12):18-21.

S30. Zhong X. *Investigation of feline herpesvirus infection in Guangdong*. Guangzhou: South China Agriculture University; 2020. 10.27152/d.cnki.ghanu.2020.000648
